# Cognitive perspectives on maintaining physicians’ medical expertise: V. Using a motivational framework to understand the benefits and costs of testing

**DOI:** 10.1186/s41235-023-00518-6

**Published:** 2023-10-10

**Authors:** Timothy J. Nokes-Malach, Scott H. Fraundorf, Zachary A. Caddick, Benjamin M. Rottman

**Affiliations:** 1 Learning Research and Development Center, University of Pittsburgh, 3420 Forbes Ave., Pittsburgh, PA 15260 USA; 2 Department of Psychology, University of Pittsburgh, 3420 Forbes Ave., Pittsburgh, PA 15260 USA

**Keywords:** Motivation, Learning, Assessment, Expectancy value, Achievement goals, Mindsets, Stereotype threat, Test anxiety

## Abstract

We apply a motivational perspective to understand the implications of physicians’ longitudinal assessment. We review the literature on situated expectancy-value theory, achievement goals, mindsets, anxiety, and stereotype threat in relation to testing and assessment. This review suggests several motivational benefits of testing as well as some potential challenges and costs posed by high-stakes, standardized tests. Many of the motivational benefits for testing can be understood from the equation of having the perceived benefits of the test outweigh the perceived costs of preparing for and taking the assessment. Attention to instructional framing, test purposes and values, and longitudinal assessment frameworks provide vehicles to further enhance motivational benefits and reduce potential costs of assessment.

## Significance

Physicians in the USA are required to take continuing education assessments at various points throughout their careers. The medical boards that administer those assessments are considering changes in their structure and implementation, including a more longitudinal assessment model. Understanding the role that motivation can play for learners in both preparing for and taking continued education assessments can inform the assessments’ design, purpose, and the policies for giving them. We take a motivational perspective on the potential benefits and costs of testing and the implications of longitudinal assessment. We review prior research on motivation for learning from cognitive, social, and educational psychology, including studies from both laboratory and classroom settings. This analysis reveals that perceived test difficulty and expectations of success, instruction framing and feedback, alignment to the values of the learner, and creating multiple lower-stakes assessment opportunities are critical issues to consider when redesigning and implementing continued educational assessments to enhance motivation, learning, and performance.

## Introduction

Physicians in the USA are required to take continuing education assessments at various points throughout their careers. Currently, many of these assessments take place every 5 to 10 years and can be viewed as summative assessments; however, many specialty boards are considering transitioning to shorter, more longitudinal assessments that can also serve as learning opportunities. This change presents an opportunity to consider some of the factors that could enhance the learning value of these assessments or otherwise make them more motivating for physicians.

In particular, for physicians to be motivated to participate in longitudinal assessments and other learning opportunities, they must view participation as having relatively high value and low costs. One general approach that can help us understand the role of value and costs in learning is the expectancy-value theory from social and educational psychology (Eccles & Wigfield, 2002, 2020; Wigfield & Eccles, 2000; Wigfield et al., 2016). This theory is widely used to explain, understand, and predict human motivation in learning and in academic performance. Expectancy-value theory posits that learners’ pursuit of an educational goal (i.e., their motivation to learn) is a function of the perceived benefits of pursuing the goal, the perceived costs of pursuing it, and the chance of succeeding if they do pursue the goal (their *expectancies*), as seen in Eq. 1 as follows:

1 $${\text{Motivation}}\;{\text{to}}\;{\text{Learn}} = {\text{Expectancies}}*\left( {{\text{Benefits}} - {\text{Cost}}} \right)$$

Thus, all other things being equal, physicians—and other learners—should be more motivated to study and practice their skills when there is a clear benefit for doing so (e.g., new knowledge, feedback on knowledge and skills, continued certification), when the costs of doing so are relatively minor (e.g., reasonable time and effort required), and when there is an expectation of success.

In this paper—part of a collection of five articles in this special issue focused on how physicians maintain medical expertise across their careers—we describe a motivational framework that builds on the expectancy-value theory that also connects to several related motivational theories and ideas from research on achievement goals, mindsets, stereotype threat, and test anxiety. We adopt the approach of a narrative review, not systematic, because we cover a wide variety of topics. To situate the strength of the evidence and claims made, we attach evidence levels (EL) to in-text citations for empirical claims (see Table 1). Evidence levels range from 1 to 6, with 1 being the strongest evidence (meta-analyses) and 6 being the weakest (opinion papers).

**Table 1:** Evidence levels for in-text citations for empirical claims

Evidence level	Type of work
1	Quantitative meta-analysis
2	Narrative review
3	Multiple original experiments/randomized controlled trials (RCTs)
4	Single original experiment (RCT)
5	Correlational or quasi-experimental study
6	Opinion paper

## Understanding motivation with situated expectancy-value theory

The most recent articulation of the expectancy-value theory has been called the *situated expectancy-value theory* (Eccles & Wigfield, 2020). This version of the theory accounts for both long-term trajectories in the development of expectancies and values as well as the short-term psychological processes engaged in task choice and performance. This version of the theory strongly emphasizes the situated nature of the factors that affect motivation, which include not only the particular features of the immediate situation and task but also the culture(s) an individual resides in, personal characteristics (e.g., gender, race / ethnicity, socioeconomic status, etc.), and past personal experiences related to the achievement activity. Viewing features of the situation as integral to the motivational processes and outcomes mirrors similar approaches in the cognitive and learning sciences that have also focused on the importance of the situation for cognitive activity and for learning and transfer outcomes (Greeno & MMAP, 1997, 1998; Lave, 1988; Lave & Wegner, 1991). We believe that the situated perspective is especially relevant to our present goal of understanding motivation in a particular situation and context—in this case, physicians’ engagement with continuing certification assessments. There are several different features of this context that are important to motivation, including both the particular prior educational and assessment experiences of physicians in the USA (e.g., attending medical school, certification tests) as well as the stereotypes associated with the profession (e.g., about who can be a physician or what resources are required to succeed).

We expand certain components of the situated theory of expectancy-value to accommodate relevant cognitive research. For example, as we elaborate upon below, previous work has focused on *expectancies of success*, but here we broaden the scope of the term and include other expectancies, such as *expectancies of being tested*, which also plays an important role in the cognitive literature on memory and learning. We highlight where there are variations—conceptual broadening or narrowing—from the situated expectancy-value concepts and features.

Further, we review and include additional motivational theories and ideas that we believe are of particular relevance to the context of physicians maintaining their expertise and completing continuing assessment. These include achievement goals (given that medical training and performance contexts often have a strong focus on mastery and performance), mindsets and stereotype threat (given varying beliefs about the profession and who can succeed), and test anxiety (given that we are focusing on continued assessment that often takes the form of high-stakes standardized tests).

Another reason to bring these different motivational ideas together into a single framework is to help further connect relevant motivational theories and ideas whose relations are often not explicitly discussed in the literature but whose features and processes often overlap and relate to one another. There have been many calls over the years to integrate these different frameworks or to compare and contrast them (e.g., Hattie et al., 2020). Although our goal here is to review those that we view as particularly relevant to physicians and the maintenance of certification, by expanding the situated expectancy-value framework to relevant cognitive research on learning and testing, we connect these strands of prior research back^1^ together in the hope that future theorizing will further integrate cognitive and motivational theories of learning and performance.

**Fig. 1:**
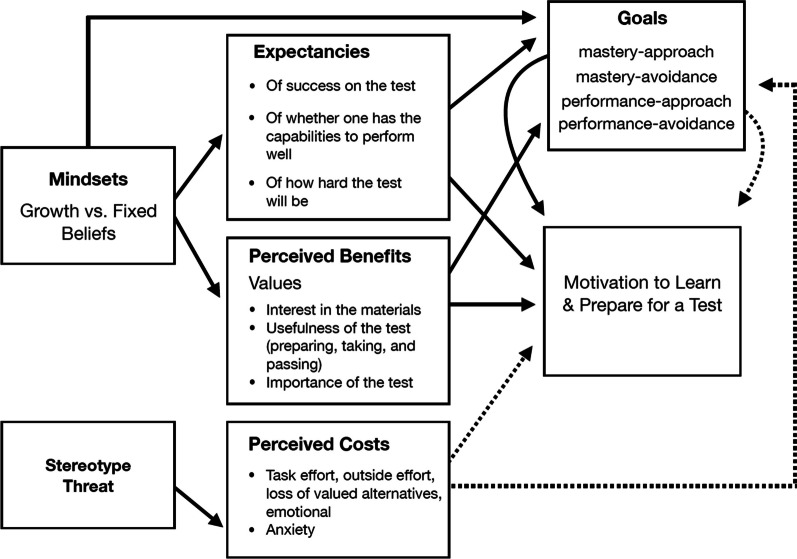
Situated expectancy-value model of the motivation to learn and interrelations to mindsets, stereotype threat, and achievement goals. The model is adapted from Eccles, Wigfield, and colleagues (Eccles & Wigfield, 2020; Wigfield & Eccles, 2000). Positive relations are denoted by the solid arrows and negative relations are denoted by dashed arrows

In Fig. 1, we present a model in which we bring together each of the motivational factors of interest, their hypothesized interrelations, and the motivation to learn. We begin by reviewing research on the effects of one’s expectancy for passing the test and of self-efficacy beliefs on engagement and learning outcomes. We then review how learners perceive the benefits of testing and explore the hypothesis that physicians will experience stronger motivation and learning to the degree that the assessment aligns with and confers value to them. We then consider related research on mindsets, which in the context of situated expectancy-value theory, can be viewed as ability beliefs that can influence expectancies and values. We also incorporate achievement goals as both a factor affected by expectancies and values as well as a mediator of the motivation to learn. Next, we discuss the potential perceived costs of testing, such as text anxiety and stereotype threat (i.e., a situation in which one is concerned about potentially confirming a negative stereotype related to an aspect of their identity), as well as approaches to mitigate those costs. We end with a discussion of directions for future work in the areas of motivation and the development of medical expertise.

## Expectancies

### Expectations of success affect how one studies and performs

A learner’s beliefs about the likelihood of success on a given task has important consequences for their learning activities. Such *expectancy beliefs* are theorized to be informed by both beliefs related to task outcome success (i.e., outcome expectancies) as well as beliefs about one’s personal capabilities to perform the task (i.e., self-efficacy; Bandura, 1997). In the situated expectancy-value model, a learner’s beliefs about the likelihood of success on a given task affects their motivation to learn. For example, if a learner is given a task that they perceive themselves as unlikely to succeed on (e.g., the test is extremely difficult or insufficient time is given), then they will be less likely to engage in that activity or to prepare adequately because they expect that they will likely fail anyway. Although high failure rates are not traditionally a problem for continuing education programs, what constitutes subjective perceptions of success and failure may be defined differently for different physicians. Prior work has shown that expectancy beliefs have a large impact on academic performance (Meece et al., 1990, EL: 5; Penk & Schipolowski, 2015, EL: 5; Priess-Groben & Hyde, 2017, EL: 5; Wigfield & Eccles, 2000, EL: 2), persistence (Scheier & Carver, 1982, EL: 4), and choice (Bong, 2001, EL: 5; Durik et al., 2006, EL: 5; Simpkins et al., 2006, EL: 5).

Conversely, high self-efficacy is associated with more productive learning behaviors (Bouffard-Bouchard et al., 1991, EL: 5; Parajes, 2008, EL: 2; Pintrich & De Groot, 1990, EL: 5; Schunk & Parajes, 2002, EL: 2). For example, students who have high self-efficacy beliefs are more likely to engage in self-regulated learning and to persist in trying to learn even in the face of difficulties or challenges (Bandura, 1997, EL: 2; Schunk & Parajes, 2002, EL: 2). These beliefs predict student retention and academic performance in school settings (Honicke & Broadbent, 2016, EL: 1) even after controlling for prior knowledge (Bailey et al., 2017, EL: 5; Kalender et al., 2020, EL: 5). A number of factors have been hypothesized to influence the development of self-efficacy, including performance feedback (e.g., test scores), observations of others, social persuasion messages, and physiological states (Bandura, 1997, EL: 2; Britner, 2008, EL: 2; Britner & Parajes, 2006, EL: 2; Usher & Pajares, 2008, EL: 2).

This research on self-efficacy has several implications for continuing assessment of medical expertise. First, an assessment must strike a balance of difficulty in that it is perceived as challenging enough to motivate constructive study activities to prepare for that test, but not so difficult that there would be no possibility for success. One way to communicate the level of difficulty is to provide representative examples of the test items to practice and receive feedback on. Second, because self-efficacy beliefs have a strong impact on how learners prepare and engage with the study materials, continuing certification programs have an opportunity to contribute to the positive development of self-efficacy. That is, the results of the assessment provide a form of performance feedback that could directly impact a physician’s self-efficacy belief (e.g., getting a higher score and thereby increasing self-efficacy). If continuing certification programs transition to more regularly spaced assessments, that provides a further opportunity to develop self-efficacy by providing multiple pieces of feedback over time. Each piece of feedback is an opportunity for an individual to adjust their appraisal of self-efficacy to be more in line with their performance (i.e., to move up or down depending on performance). Providing repeated performance feedback creates an opportunity to determine whether such feedback leads to more accurate self-assessment.

### Expectations of test difficulty affect engagement and performance

While the expectancies component of the situated expectancy-value model focuses on expectancies for success on a task, as we discuss above, we also consider here how the expectation that one will be tested in the future can itself create the opportunity to engage in productive study and learning activities. Although just knowing about the existence of an upcoming assessment does not necessarily promote learning (Hyde & Jenkins, 1973, EL: 4; Postman, 1964, EL: 3), an expectation of being tested in a particular way or on certain types of material can lead to better learning (McDaniel et al., 1994, EL: 3; Szpunar et al., 2007, EL: 4). The anticipated difficulty of the test matters, too. For example, laboratory researchers have often examined test difficulty by contrasting a *recall* task, in which learners must bring to mind the required information (e.g., a fill-in-the-blank item or essay), with a *recognition* task, in which learners must merely identify the information when it is presented (e.g., multiple-choice or true–false items). All other things being equal, recall is more difficult than recognition. Thus, people expecting a difficult recall test learn and remember more than people expecting an easier recognition test, regardless of the type of test they actually receive (Balota & Neely, 1980, EL: 4; Connor, 1977, EL: 3; d’Ydewalle et al., 1983, EL: 4; Hall et al., 1976, EL: 3; Leonard & Whitten, 1983, EL: 3; Maisto et al., 1977, EL: 4; Neely & Balota, 1981, EL: 4; Schmidt, 1988, EL: 4; c.f., Finley & Benjamin, 2012, EL: 3).

What accounts for this *test expectancy effect*? Benefits of intentional encoding (i.e., with the goal to learn) appear to be driven largely by the activities that learners engage in when preparing for a test (Hyde & Jenkins, 1969, EL: 4; Hyde & Jenkins, 1973, EL: 4; c.f., Neely & Balota, 1981, EL: 4). Learners expecting a more difficult test can engage in more effective study behaviors, such as studying longer (d’Ydewalle et al., 1983, EL: 4; Thiede, 1996, EL: 3), continuing to practice after an initial quiz (Szpunar et al., 2007, EL: 4), and/or engaging in deeper, more meaningful practice (Hall et al., 1976, EL: 3; Leonard & Whitten, 1983, EL: 3; Schmidt, 1988, EL: 4). Conversely, even offering financial incentives does not increase learning when learners are required to use ineffective learning strategies (Craik & Tulving, 1975, EL: 4).

The results reviewed above suggest that physicians learn and retain more when they expect to be tested on the knowledge and skills they are developing. This is especially the case when the perceived difficulty of the test is difficult enough^2^ to engender deeper, more effective preparation and when the environment guides physicians to effective study behaviors and activities to capitalize on their increased motivation.

## Perceived benefits: what is the value of the test to the learner?

In the situated expectancy-value model, the perceived value of a given task or assessment plays a critical role in motivation to prepare for and engage with it. Value is hypothesized to consist of three distinct components, each of which we review in turn (Wigfield et al., 2016).

### Intrinsic task value

*Intrinsic task value* is interest in a task or assessment for its own sake. Theories of interest typically discuss two different kinds: situational and individual (Hidi & Harackiewicz, 2000; Hidi & Renninger, 2006; Krapp, 1999; Schiefele, 1991). *Situational interest* is hypothesized to be a momentary experience that is driven by environmental factors (e.g., a loud noise) and correlated with both cognitive (e.g., attention) and affective (e.g., surprise) factors (Hidi & Harackiewicz, 2000, EL: 2). *Individual interest* is hypothesized to be a longer-lasting engagement and is associated with one’s knowledge, values, and feelings about the particular topic or task (Renninger, 2000, EL: 2). One perspective on how intrinsic task value emerges is given by discrepancy theory, which posits that learners are motivated to increase a valued competency when they perceive a *discrepancy* between their current skill and a given goal or standard (Fox & Miner, 1999; see also regulatory focus theory, Higgins, 1997, 2012). Laboratory studies have confirmed that people are sensitive to gaps between perceived and desired knowledge (Dunlosky & Hertzog, 1998, EL: 5; Son & Metcalfe, 2000, EL: 3; Tullis & Benjamin, 2010, EL: 2).

Much prior work has shown that individual interest in the task can increase self-reported effort (Renninger & Hidi, 2002, EL: 5), positive self-regulation (O’Keefe & Linenbrink-Garcia, 2014, EL: 5; Renninger & Hidi, 2019, EL: 2), and deep strategy use (Schiefele et al., 1995, EL: 5). It is also associated with better grades in school (Harackiewicz et al., 2008, EL: 5; Schiefele et al., 1992, EL: 1). Intrinsic task value may also be linked to achievement goals for a particular task, as we elaborate upon below. In the domain of medicine, medical students’ interests and perceived competence have been shown to predict important career choices, such as medical specialty decisions (Williams et al., 1997, EL: 5). Other research has shown that experimental interventions to increase intrinsic task value can facilitate interest and subsequent learning. For example, testing with feedback, in addition to directly enhancing learning, can also increase the desire to learn more about a topic (Abel & Bäuml, 2020: EL 3). This finding aligns with discrepancy theory in that learners need to become aware of (i.e., perceive) discrepancies between actual and desired knowledge to become highly motivated.

Interest and task performance can also be increased by personalizing content (Bernacki & Walkington, 2018, EL: 4; Walkington & Bernacki, 2018, EL: 2). This research implies that the more the content of the test (e.g., topics and patient scenarios) matches the interests of the physician, the more motivated they will be to learn and keep current. It would be desirable to collect information on physicians’ medical interests and personal practice to match those interests, or perhaps to allow physicians some opportunities to select relevant topic areas or problem scenarios to be tested on. Another technique to potentially help select material in a longitudinal spaced-repetition paradigm would be to collect ratings of relevance and use them to prioritize content which information is re-presented.

### Utility value

Another aspect of value is called *utility value*, or the degree to which preparing for and taking the test is useful for accomplishing some valued outcome; that is, as a means to an end (Eccles, 2009; Wigfield & Eccles, 2002). Often, these valued outcomes are broader personal, educational, or professional goals. Correlational research has shown that utility value is positively associated with engagement, learning, and performance outcomes, such as higher grades (Harackiewicz et al., 2016, EL: 2; Harackiewicz et al., 2014, EL: 2). Further, intervention studies have shown that, when utility value increases, so does academic performance (Harackiewicz & Priniski, 2018, EL: 2; Harackiewicz et al., 2016a, 2016b, EL: 4, Hulleman et al., 2010, EL: 4).

Utility value is relevant to longitudinal assessment of medical expertise in at least two ways. The first concerns the usefulness of preparing to take the assessment. That is, does a physician view preparing for the assessment as a helpful activity that contributes to their medical training and skill development more generally, or just something they do because they have to? The more a physician sees connections between the activities of studying and their broader professional goals (e.g., acquiring critical new knowledge), the more motivated they will be to study. The second concerns the value ascribed to the test itself. That is, does a physician view the test as useful to achieving broader educational goals (e.g., staying current) and professional goals (e.g., staying employed, being promoted)?

Interviews with physicians preparing for and taking high stakes tests show a range of perceptions of how relevant and related the content is to their current practice (Chesluk et al., 2019a, 2019b, EL: 5). The work we reviewed above implies that such variation in perceptions is likely to affect physicians' motivation to learn. If physicians see the activity of studying between longitudinal assessment sessions as relevant to their broader professional goals, they will be more motivated and more deeply engaged with the material. Alternatively, if they view the assessment as unrelated and disconnected, they may engage only superficially. Fortunately, some evidence suggests that utility value is amenable to intervention; for instance, in academic settings, it can be improved by having the learner briefly write about the usefulness of the class or discipline to them (Harackiewicz & Priniski, 2018, EL: 2). Feedback within an assessment can also promote utility value. Some longitudinal assessment platforms require the participant to rate each question’s relevance to their medical practice. Periodically providing feedback (e.g., as summary feedback between assessment sessions) regarding questions that a learner missed but that they also rated as relevant to their practice may provide additional motivation for them to review those concepts.

### Attainment value

The third component of value is *attainment value*, or the importance of doing well on a given task or assessment. In the current context, attainment value would capture how important it is to the individual to prepare for the assessment and perform well on it. This judgment will depend on the physician’s perception of what the assessment measures (e.g., relevant medical knowledge and skills), how accurately it measures those competences, and the ramifications of passing or failing the assessment (e.g., often required for preferred employment).

Attainment value is theorized to have implications for one’s self-concept and identity (Eccles, 2009; Eccles & Wigfield, 2020; Ryan & Deci, 2020). For example, self-determination theory implies that performance outcomes provide “data” that can be used to confirm or deny aspects of one’s identity (La Guardia, 2009; Ryan & Deci, 2020), including three core needs of autonomy, relatedness, and—most critical to our purposes—competence. If one perceives the assessment as measuring critical medical competence and performs well, that result can be interpreted as confirming one’s view of oneself as a competent, expert physician. Alternatively, if one perceives the assessment as important but performs poorly on it, it could call into question either one’s view of oneself as an expert, knowledgeable physician, or the validity and accuracy of the test.

Attainment value has been shown to be positively related to engagement (Putwain et al., 2019, EL: 5), effort (Guo et al., 2016, EL: 5), self-concept (Arens et al., 2019, EL: 5), and academic achievement (Trautwein et al., 2012, EL: 5; Meyer et al., 2019; EL: 5). Laboratory research on memory and learning also supports the relevance of attainment value. In one lab paradigm, each to-be-learned item is experimentally assigned a point value that learners are awarded for successful retention, and learners are tasked with earning as many points as possible. Learners consistently remember more of the high-value items, demonstrating that value guides priorities for learning and retention (Castel et al., 2002, EL: 3; Castel et al., 2011, EL: 4; Castel et al., 2013, EL: 3; Hennessee et al., 2018, EL: 3; McGillivray & Castel, 2017, EL: 3).

This work implies that physicians’ perception of the importance of the task and test affects their motivation to learn. The more that physicians see the test as measuring an important set of skills and knowledge, the more time and effort they will invest in performing well on the test. Further, if the assessment provides feedback relevant to physicians’ self-concepts and identities (e.g., their identity as a skilled medical doctor), they will show higher investment in developing their skills and performing well on the assessment. Lastly, longitudinal assessments of medical expertise could encourage physicians to learn and retain particular skills by assigning them higher value or by apportioning more questions to these topics within the assessment (as is often already done).

## Growth mindsets promote motivation and learning

Another important motivational factor that can impact how learners prepare for and engage with assessments is their mindset and beliefs about ability. *Mindset* is a broad term used to describe a set of beliefs that can impact one’s expectations, meaning-making, and behaviors (Dweck & Yeager, 2019, EL: 2).

One of the most powerful mindsets that has been investigated is people’s beliefs about intelligence. Within the situated expectancy-value model of Eccles and Wigfield (2020), mindsets regarding intelligence are captured as part of the self-concept of one’s abilities that can influence expectancies and values. Carol Dweck and her colleagues have been some of the leading researchers on mindsets about intelligence and have focused on two types of beliefs. The first is a belief that intelligence is malleable and can change with experience in a domain, which has been called a *growth mindset*. The other is a belief that intelligence is inherited and cannot be changed through experience, which has been called a *fixed mindset*. Intelligence mindsets and ability beliefs have been hypothesized to affect a learner’s expectancies, values, and achievement goals, which in turn affect the motivation to learn (Fig. 1). For example, a growth mindset is hypothesized to lead to positive self-regulated learning behaviors, such as effort in the context of challenge, which in turn lead to better learning and achievement outcomes (Blackwell et al., 2007, EL: 4&5).

A growth mindset predicts positive academic achievement (Costa & Faria, 2018, EL: 1; Blackwell et al., 2007, EL: 4; Gunderson et al., 2013, EL: 5, Henderson & Dweck, 1990, EL: 2, Paunesku et al., 2015, EL: 4; cf. Li & Bates, 2019, EL: 4). Growth and fixed mindsets also relate to students’ self-reported interest (Haimovitz et al., 2011, EL: 5), effort (Blackwell et al., 2007, EL: 5; Miele et al., 2011, EL: 5; Miele & Molden, 2010, EL: 3), and learning goals (Blackwell et al., 2007, EL: 5; Haimovitz et al., 2011, EL: 5). For example, at a correlational level, a growth mindset during the middle-school years predicts learning goals (e.g., “An important reason why I do my school work is because I like to learn new things”) and positive effort beliefs (e.g., “The harder you work at something, the better you will be at it”), which in turn predict positive study strategies (e.g., “I would spend more time studying for tests”) and performance (e.g., achievement test scores) (Blackwell et al., 2007, EL: 5). There is some evidence that the link between growth mindset and academic achievement is causal: Interventions designed to promote growth mindsets, with messages that portray intelligence as malleable with experience and training, lead to positive changes in motivational and achievement outcomes (Blackwell et al., 2007, Expt. 2, EL: 4; Mueller & Dweck, 1998, EL: 4; Yeager, et al., 2016, EL: 4; c.f. Li & Bates, 2019).

In sum, mindsets about intelligence can have powerful downstream effects on motivational and learning outcomes and can directly impact expectancies, values, and goals. Thus, physicians who believe their intelligence and skills are malleable may be more likely to adopt good learning behaviors and goals, which would further their retention of cognitive skills. Physicians’ adoption of a growth mindset may be fostered by the shift in continuing certification toward more regular spaced testing, which provides the opportunity to improve over time.

## Achievement goals and the benefits of pursuing mastery

*Achievement goals* are the reasons why people engage in study and test activities. Achievement goals are sometimes described and investigated separately from expectancy-value theory, but sometimes are included in an overarching model (Plante et al., 2013). In the situated expectancy-value model of Eccles and Wigfield (2020), long- and short-term goals are described as factors that can influence expectancies for success and subjective task values. Others have hypothesized the converse: that expectancies and values affect the adoption of achievement goals (Elliot, 1999; Greene et al., 2004). Some empirical work supports this second view by showing that expectancies and values have both direct effects on motivation for learning *and* indirect effects through achievement goals (Plante et al., 2013, EL: 5). We incorporate this second view into our model depicted in Fig. 1.

Achievement goals can either be *mastery-oriented*, with a focus on improving and understanding the material in comparison to one’s prior understanding, or *performance-oriented*, with a focus on demonstrating ability in comparison to others (Dweck, 1986; Elliot, 1999). Each of these two goals can be approach-or avoidance-based (Elliot, 1999). *Approach-based* goals are defined by striving toward a positive outcome, and *avoidance-based* goals are defined by avoiding negative outcomes. Combining these different dimensions results in four different goals: a *mastery-approach* goal to learn as much as possible, a *mastery-avoidance* goal to avoid loss of knowledge or skills, a *performance-approach* goal to perform better than others, and a *performance-avoidance* goal not to perform worse than others (Elliot & McGregor, 2001; Elliot & Murayama, 2008). We thus view achievement goals as particularly relevant for the context of developing and maintaining medical expertise given the focus on mastery and performance in training and assessment.

Although there has been little work specifically examining achievement goals in the context of practicing physicians, many laboratory experiments and classroom studies have examined these four achievement goals in relation to engagement, learning, and performance outcomes. This literature has consistently linked performance-avoidance goals to negative outcomes, such as poor performance (e.g., grades and tests), as well as low self-efficacy, poor study habits, and procrastination (Elliot & Church, 1997, EL: 5; Elliot & McGregor, 1999, EL: 5; Elliot et al., 1999, EL: 5). In contrast, mastery-approach goals have been consistently associated with positive outcomes, such as self-reported interest and engagement (Elliott & Dweck, 1988, EL: 4; Elliot et al., 1999, EL: 5; Harackiewicz et al., 2002a, 2002b, EL: 5; Harackiewicz et al., 2008, EL: 5) and learning and transfer (Belenky & Nokes-Malach, 2012, 2013, EL: 5). The fact that mastery-approach goals have been related to knowledge transfer is promising for learning in medical education contexts in that it may help physicians acquire sought-after skills critical to adaptive medical expertise (Mylopoulos et al., 2018, EL: 2).

Performance-approach goals correspond to a more intermediate level of performance; they have been related to some positive outcomes, such as better grades and exam performance (Harackiewicz et al., 2002a, 2002b, EL: 2; Linnenbrink-Garcia et al., 2008, EL: 2), but also some negative outcomes, such as less effective self-reported study behaviors (i.e., rote memorization) (Midgley et al., 1996, EL: 5; Senko et al., 2011, EL: 2).

Lastly, mastery-avoidance goals have been the least studied of the four goal types but may be particularly relevant to certification boards as they pertain to avoiding the loss of knowledge and skills that were previously mastered. These goals have been associated with mixed results (Hulleman et al., 2010, EL: 4; Linbrink et al., 2008, EL: 2), including both positive outcomes, such as learning (Richey & Nokes-Malach, 2013, EL: 3), and negative outcomes, such as self-reported test anxiety (Elliot & McGregor, 2001, EL: 5).

How do learners come to adopt one type of achievement goal or another? A number of factors can influence achievement goals (Ames, 1992, EL: 2). Prior experimental and classroom work has shown that instructions can affect the goals that learners adopt in the moment (Elliot & Harackiewiz, 1996, EL: 3; Elliot & Dweck, 1988, EL: 4; Graham & Golan, 1991, EL: 3). For example, telling students that the purpose for a given task is either to “develop their ability or skill and learn from mistakes” or conversely “to compare ability to others and to determine whether they are better or worse than others” can impact learning outcomes and task engagement (Bereby-Meyer & Kaplan, 2005, EL: 3; Elliot & Harackiewicz, 1996, EL: 3). Other work has shown that the type of task can also impact the types of goals adopted. For example, a *discovery task*, in which the learner aims to find a principle that explains a data pattern, has been shown to promote the adoption of mastery-approach goals relative to a task presented as direct instruction followed by practice (Belenky & Nokes-Malach, 2012, EL: 4). The framing of the task is particularly relevant for the continuing certification of medical expertise because the instructions could easily be written to facilitate the adoption of a mastery goal. For example, physicians could be asked to focus on developing their understanding and trying to improve their score over time—aiming to achieve their personal best.

## Perceived costs of testing

### General aspects of psychological cost

In the situated expectancy-value framework, an important subcomponent of the motivation to learn is the perceived cost of the study activity or test. This component of the model has historically received less attention than expectancy and other aspects of value^3^ (i.e., intrinsic, utility, attainment); however, more recently, several efforts have been made to develop measurement tools that capture important aspects of cost (Conley, 2012; Flake et al., 2015; Trautwein et al., 2012) and to better understand its role in the expectancy-value framework (Barron & Hulleman, 2015; Eccles & Wigfield, 2020). Four aspects of cost have been identified (Flake et al., 2015). *Task effort* refers to the amount of time and energy of performing a task itself. *Outside effort* refers to the amount of time and energy required for other tasks than the focal task (e.g., family and work obligations), which may result in the perception of not having enough time to dedicate to the focal task. The *loss of valued alternatives* refers to what one has to give up to prepare for the task or test. For example, in the current context, a valued alternative lost in preparing for continuing certification program assessments may be leisure time or family time (Galla et al., 2015: EL 5; Kurzban et al., 2013). The last aspect is *emotional cost*, which refers to the potential stress and worry caused by the task. For example, anxiety in anticipation of a high-stakes test would increase the perceived emotional cost of the test, as we discuss in further detail below. In interviews with physicians about how they prepared for and took continuing certification examinations, the lack of time available because of outside effort involved in studying and the loss of valued alternatives emerged as important themes (Chesluk et al., 2019a, 2019b, EL: 5).

In the situated expectancy-value model, the more of these perceived costs, the less likely one is to be motivated to learn and prepare for the test. Prior work has shown that perceptions of cost predict additional variation in motivation and performance outcomes above and beyond expectancies and values (Jiang et al., 2018; EL 5; Perez et al., 2014; EL 5). In principle, then, the more that perceived costs can be reduced, the stronger an individual’s motivation to learn. A recent intervention that aimed to reduce cost in an introductory physics course by focusing on the normalization and temporary nature of effort costs has shown some promising results in reducing subsequent perceived costs and increased class performance (Rosenzweig et al., 2020, EL: 4; c.f. Rosenzweig et al., 2022, EL: 4). This work suggests that one way to mitigate the perceived costs of testing would be to discuss those costs in advance in an effort to normalize them.

Another aspect of continuing certification programs that may impact one’s perceptions of cost are the monetary costs associated with certification. There is no research that we know of that has investigated the impact of financial cost of tests on perceived costs within the situated expectancy-value framework, but this could be a useful direction for future work.

### Test anxiety

As we discuss above, one form of cost in the situated expectancy-value is emotional cost, which includes anxiety. Indeed, there is a substantial literature specifically on test anxiety, which we review here because it is relevant to the context of continued assessment. *Test anxiety* is a multi-faceted construct consisting of physiological, psychological (e.g., emotional, cognitive), and behavioral components (Zeidner, 1998, 2009; von der Embse, 2018). It is hypothesized to emerge as worries or fear about a negative evaluation in relation to an evaluative test. Several mechanistic models of test anxiety have been proposed and tested over time, including interference (Alpert & Haber, 1960, EL: 5; Liebert & Morris, 1967, EL: 5), deficit (Tobias, 1985, EL: 2), and transactional models that incorporate components of the former two (Spielberger & Vagg, 1995, EL: 2; see von der Embse, 2018, EL: 1 for a review). More recently, biopsychosocial models have been proposed that focus on the interactive relations between biological, psychological, and social/environmental factors that trigger test anxiety in-the-moment (Segool et al., 2014, EL: 5; Jamieson, 2017, EL: 2).

Although some arousal may be good, many individuals approach standardized tests with levels of anxiety that are high enough to impair performance (von der Embse, 2018, EL: 1). Famously, the Yerkes-Dodson law of arousal and performance states that a moderate level of arousal leads to optimal performance (Yerkes & Dodson, 1908, EL: 3). This “inverted U” model predicts poor performance at low levels of arousal because one is not adequately alert or engaged with the task and at high levels of arousal because one may experience anxiety and worry that interfere with performance. High levels of arousal, anxiety, and worry have been investigated broadly across physical skills and performances as well as intellectual and academic contexts (Alpert & Haber, 1960, EL: 5; Beilock & Carr, 2001, EL: 3; Beilock et al., 2017, EL: 2; Mandler & Sarason, 1952, EL: 4; Sarason, 1980, EL: 2). Test anxiety, in particular, is associated with poorer performance on classroom tests, GPA, IQ tests, and standardized tests (Ackerman & Heggedstad, 1997, EL: 1; Hembree, 1988, EL: 1; von der Embse, 2018, EL: 1).

One reason that high levels of anxiety may be harmful to test-taking is that anxiety can reduce working memory resources (Beilock, 2008, EL: 2; Beilock & Carr, 2005, EL: 4; Moran, 2016, EL: 1). Moran (2016, EL: 1) examined the relationship between self-reported anxiety and working memory capacity in a meta-analysis (*N* = 22,061 participants) and found a small to moderate negative relationship (Hedges’ *g* = − 0.33). However, there is still much debate about the boundary conditions of the relations and the exact mechanisms at play. One hypothesis is that anxiety impairs performance via multiple routes: worries impair verbal processing, and high arousal impairs spatial storage (Moran, 2016, EL: 1).

The deleterious effects of anxiety may intensify in response to high-pressure tests. Hinze and Rapp (2014, EL: 3) found that the benefits of testing for learning were diminished if there was significant performance pressure during episodes of memory retrieval. These findings illustrate the importance of reducing pressure during testing in order to maximize learning outcomes. One method that Hinze and Rapp found could reduce pressure was to weigh earlier retrieval-practice questions less than later questions so that learners who do poorly early on can identify areas of weakness and improve upon them. Other work has found that practicing for an assessment using retrieval practice can reduce test anxiety (Agarwal et al., 2014, EL: 3). Thus, the development of a longitudinal assessment focused on learning benefits may reduce the perceived pressure of a one-time, high-stakes test.

### Stereotype threat

In the situated expectancy-value framework, stereotypes are described as operating in a broader cultural milieu that is theorized to impact subsequent self-perceptions and task expectancies and values. Here, we elaborate on these effects and connect them to the broader literature on stereotype threat, another phenomenon that can be triggered in high-stakes testing.

*Stereotype threat* refers to the diminished performance that can occur when reminded of a negative stereotype in a domain in which one otherwise identifies and has high competence (Steele, 1997). It has been observed in varying populations and domains, including women in math, Black Americans in higher education, White males in sports, and older adults in their episodic memory (Barber & Mather, 2013: EL: 3, Bouazzaoui et al., 2020, EL: 5; Nguyen & Ryan, 2008, EL: 1; Rahhal et al., 2001: EL: 3; Steele & Aronson, 1995, EL: 5; Stone et al., 1999, EL: 5).

Stereotype threat is thought to occur because actors of a stereotyped group may exhibit poorer performance as a consequence of not wanting to reinforce the stereotype. Multiple mediating mechanisms have been proposed for this underperformance, including the depletion of working memory resources being consumed to suppress negative thoughts, interference from attending to cognitive processes that are typically automatic, and strategies to protect one’s self-concept (e.g., self-handicapping), among others (Spencer et al., 2016, EL: 2). Shewach et al., (2019, EL: 1) found that when motivational incentives, such as a monetary reward, are present, stereotype threat is much less pronounced than when they are absent (Cohen’s *d*s 0.14 vs. 0.41, respectively), suggesting that motivation plays an important role in stereotype threat.

Stereotype threat is likely to be applicable to physicians in a continuing assessment given that this context meets the criteria of a domain with which the learners identifies and has high competence (Steele, 1997). Physicians are likely to be highly identified with the medical domain and view themselves as having high competence given their extensive education and training; further, the context of continuing assessment may be viewed as providing results that bear on their evaluation of that competence. However, there are also negative stereotypes associated with particular medical subdisciplines regarding social identities of gender (Fassiotto et al., 2018: EL: 5; Myers et al., 2020: EL: 4) and race and ethnicity (Bullock et al., 2020: EL: 5).

Given the evidence for stereotype threat in testing, a few recommendations for longitudinal assessments may be beneficial. First, stereotype threat is more likely to occur when a test is more difficult (Nguyen & Ryan, 2008, EL: 1; Shewach et al., 2019, EL: 1). Thus, framing any longitudinal assessment in terms of its learning benefits may serve to lower anxiety and reduce perceptions of a test as “high-stakes” (see “reconstrual interventions” in Spencer et al., 2016; EL: 2). Second, in situations where demographic information must be collected, this should occur *after* any testing or at some other time not directly before the assessment to reduce potentially activating negative stereotypes related to the individuals’ demographics. Third, testing materials should include a diverse cast of characters and should be carefully reviewed so as not to reinforce stereotypes through the testing content.

## Relations between features of the model

The situated expectancy-value framework theorizes that relations between expectancies and values have multiplicative effects on motivation and performance. That is, these components are not just additive but interact with one another. Some prior empirical work has found evidence to support this view in that interactive effects of measures of expectancy and value predicted additional variance in motivation and performance above and beyond expectancy and value alone (Nagengast et al., 2011: EL: 4; Trautwein et al., 2012: EL 4). However, much remains to be discovered as to how expectancy and value interact with one another and with cost.

## Proposed studies and future directions

### Measuring motivation

There is a lack of research on the effects of motivation in longitudinal testing scenarios, including continuing certification programs. One reason for this may be that, to measure motivation in this context, one must determine not only *what* components of motivation to measure but *how* to measure them.

There are many potential methods to measure aspects of motivation, including self-report surveys, interviews, behaviors (e.g., choice, time spent, errors, etc.), and physiology, among others. We suggest beginning with self-report surveys because researchers have developed validated measures for many of the constructs that we have discussed that can be used in a variety of educational contexts (e.g., self-efficacy, Bandura, 2006; Fencl & Scheel, 2005; interest and value, Linnenbrink-Garcia et al., 2010; Pintrich et al., 1993; cost, Flake et al., 2015; achievement goals, Elliot & Murayama, 2008; mindsets, Dweck, 1999, 2006; test anxiety, Putwain et al., 2021). Self-report surveys are also relatively easy to implement in a longitudinal assessment context. A first step, then, would be to adapt the items to the continuing certification context and conduct validation studies with this new domain and population.

Given such validated instruments, there are many research questions that could then be investigated concerning the role of motivation in longitudinal assessments. We suggest that one place to start would be to assess whether the motivational components reviewed in this paper—expectancies, perceived values and costs, achievement goals, and mindsets—predict performance in the assessments. Measuring these components of motivation would contribute to basic science by characterizing motivation in the medical field and, more broadly, in an area where individuals have much more expertise in a domain than more novice populations. Such data would be relevant to theory testing and generalization and to understanding relations among motivational constructs. They could also be extremely informative in assessment design decisions and potential interventions. For example, if self-reported utility value strongly predicts performance in the continuing certification context, then interventions (e.g., a brief writing task or instructional framing) could be tested to increase perceptions of utility value, as we elaborate upon below.

In this context, motivation would be relevant both as a process as an outcome. Whether a particular motivational factor, such as utility value, predicts performance in the assessment may shine light onto the specific motivational processes at play when one is taking the assessment. In addition, if it is determined that certain motivational components are particularly important in this context, then measures of motivation could also serve as outcomes or dependent measures for other interventions and assessment changes.

### The role of financial cost in a situated expectancy-value framework

As we noted above, there is little or no work examining how the financial cost of taking an assessment may affect motivation, though from the learner’s perspective, these may be an important consideration. Future work should examine how financial costs relate to other cost perceptions and how they affect motivation and performance outcomes.

### Interventions to increase motivation and performance

Throughout our review, we identified multiple components of our motivational framework for which past work has developed successful interventions. Such interventions can enhance aspects of value, facilitate productive goals and mindsets, and mitigate potential costs. Here, we describe five that may be particularly well suited to the current context of longitudinal assessments.

Interventions that introduce choice and personalization can increase intrinsic task value and performance (Walkington & Bernacki, 2018, EL: 2; Patall et al., 2008, EL: 1). Thus, allowing individuals some choice of areas to be tested on could increase interest and engagement. Similarly, we hypothesize that personalizing the test to the individual taking it—matching test items to the context or contents of interest—would increase engagement, preparation, and performance outcomes. This would not require any choice within the assessment system itself; the assessment system could automatically assign personalized test content based on an initial survey that the physician would take about their clinical practice.

Second, it might also be possible for interventions to increase the assessment’s perceived utility value. For example, we would predict that motivation to learn could be increased by a 10-min exercise in which individuals write about how the preparation and assessment is relevant to their educational and professional goals.

Third, building on interventions in the broader achievement goal literature (Elliot & Harackiewiz, 1996, EL: 3; Elliot & Dweck, 1988, EL: 4; Graham & Golan, 1991, EL: 3), an assessment’s instructions and structure could help individuals adopt a mastery achievement goal. For example, instructions could emphasize understanding and improvement. The shift toward longitudinal assessment also allows for a focus on *intrapersonal comparison*; that is, focus on how a physician can improve relative to their past performance rather than other physicians.

Fourth, revising instructions may also provide an avenue to reduce some aspects of perceived costs. Instructions that normalize aspects of the time and effort required to successfully prepare and engage in the longitudinal assessments may reduce perceived cost.

Lastly, discrepancy theory (Fox & Miner, 1999) offers a method for measuring and instilling motivation that, although originally proposed for other forms of continuing medical education, can readily be adapted for longitudinal assessment programs. First, physicians subjectively rate aspects of their clinical competency (e.g., knowledge of diabetes) and the desirability of those aspects (e.g., how important to you is it to have expert knowledge about diabetes?). Then, they take an assessment of the target competency. Finally, a discrepancy score between perceived and actual competency is computed and presented. Physicians who value a particular target competency, but who were unaware of a gap between their perceived and actual ability, may gain an intrinsic desire to improve—especially in the context of a longitudinal assessment program, where they could focus on those topics when studying for the next assessment.

## Summary and conclusion

In this paper, we took a situated expectancy-value approach to thinking about the role of motivation in continuing certification programs. We reviewed basic motivational theory and empirical work from laboratory and classroom settings, and we discussed their implications and applications in the context of continuing certification program assessments. This review suggests several motivational benefits of testing as well as some potential challenges posed by high-stakes standardized tests.

Many of the motivational benefits for testing can be understood from the equation of having the perceived benefits of a test outweigh the perceived costs of preparing for and taking it. We found that a sufficiently challenging test can increase both motivation to learn and later performance as long as the test is not perceived as *too* difficult; that is, if learners perceive that investing effort is likely to increase success on the test. Two ways to make clear the level of difficulty are to describe the specific task items used and to give representative problems to practice and receive feedback on.

We also reviewed three components of value (intrinsic, utility, and attainment) that should be attended to when designing an assessment. The ideal assessment should be perceived as relevant to the practitioner’s interests (e.g., in terms of the topics and scenarios). It should be useful to furthering the practitioner’s educational and professional goals, such as developing expertise and staying current. And, it should be perceived as important; that is, as an accurate measure of medical knowledge and skills and as an opportunity to confirm a physician’s identity as a skilled medical expert. These values can be highlighted in the instructions and framing of the assessment and potentially in preparatory activities that might further reinforce them (e.g., a writing activity to discuss why this assessment is helpful to one’s goals). Similarly, framing the longitudinal assessment and feedback as an opportunity to learn and develop can further facilitate the adoption of mastery-approach goals and growth mindsets.

Complementing efforts to boost perceived value is an effort to mitigate perceived costs. It would be helpful to convey the task effort as reasonable and worthwhile. High-stakes assessment can also carry an emotional cost in the form of test anxiety, but the move to a longitudinal assessment scheme of more frequent testing may reduce test anxiety relative to less frequent, higher-stakes tests. We also discussed the related phenomenon of stereotype threat, which can be mitigated by emphasizing the assessment as an opportunity to improve (as opposed to a high-stakes, evaluative test), highlighting the components of value previously described, asking demographics at the end of the assessment or at some other time not right before the assessment, and including diverse demographic features in the testing clinical scenarios.

By considering how both motivational and cognitive factors relate to the benefits and costs of longitudinal assessment, future work can build theories that integrate across these frameworks and a practical opportunity to design multi-purpose assessments that are both engaging and useful.

## Data Availability

Not applicable.

## Footnotes

1 Work on learning and motivation at the beginning of the twentieth century by Thorndike, Hull, and Tolman, among others, closely connected the processes of motivation and learning together within singular theories (see Klein, 2019, for an overview). We hope that future work continues to bring research on cognition and learning and motivation and learning back together in integrated ways.

2 Of course, we discuss above, if the test is expected to be *too* difficult and outcome expectations are consequently low, learners may instead disengage from the task entirely.

3 In the situated expectancy-value framework, cost is considered a subcomponent of value. Here, we represent cost separately from value in Fig. 1 to highlight its role and to both build on the prior work on costs as well as broaden the definition to consider other types of costs (e.g., financial costs of taking the assessment).
